# Authentication of the Origin, Variety and Roasting Degree of Coffee Samples by Non-Targeted HPLC-UV Fingerprinting and Chemometrics. Application to the Detection and Quantitation of Adulterated Coffee Samples

**DOI:** 10.3390/foods9030378

**Published:** 2020-03-24

**Authors:** Nerea Núñez, Xavi Collado, Clara Martínez, Javier Saurina, Oscar Núñez

**Affiliations:** 1 Department of Chemical Engineering and Analytical Chemistry, University of Barcelona, Martí i Franquès 1−11, E08028 Barcelona, Spain; xavi.collado08@gmail.com (X.C.); clmartig28@alumnes.ub.edu (C.M.); xavi.saurina@ub.edu (J.S.); 2 Research Institute in Food Nutrition and Food Safety, University of Barcelona, Recinte Torribera, Av. Prat de la Riba 171, Edifici de Recerca (Gaudí), Santa Coloma de Gramenet, E08921 Barcelona, Spain; 3 Serra Húnter Fellow, Generalitat de Catalunya, Rambla de Catalunya 19-21, E08007 Barcelona, Spain

**Keywords:** HPLC-UV fingerprinting, non-targeted analysis, coffee, food authentication, food adulteration, principal component analysis (PCA), partial least squares regression (PLSR)

## Abstract

In this work, non-targeted approaches relying on HPLC-UV chromatographic fingerprints were evaluated to address coffee characterization, classification, and authentication by chemometrics. In general, high-performance liquid chromatography with ultraviolet detection (HPLC-UV) fingerprints were good chemical descriptors for the classification of coffee samples by partial least squares regression-discriminant analysis (PLS-DA) according to their country of origin, even for nearby countries such as Vietnam and Cambodia. Good classification was also observed according to the coffee variety (Arabica vs. Robusta) and the coffee roasting degree. Sample classification rates higher than 89.3% and 91.7% were obtained in all the evaluated cases for the PLS-DA calibrations and predictions, respectively. Besides, the coffee adulteration studies carried out by partial least squares regression (PLSR), and based on coffees adulterated with other production regions or variety, demonstrated the good capability of the proposed methodology for the detection and quantitation of the adulterant levels down to 15%. Calibration, cross-validation, and prediction errors below 2.9%, 6.5%, and 8.9%, respectively, were obtained for most of the evaluated cases.

## 1. Introduction

The quality of natural products is an issue of great interest in our society. Taking into account the food chain complexity and the various factors involved between food production and consumption, food handling and adulteration practices are increasing. In this sector, fraud is defined as a form of conscious deception about the quality of a food product for profit, and adulteration is defined as the partial replacement of food of certain quality by another similar with lower quality and price. Adulteration and manipulation of foods to fool final consumers is illegal worldwide, not only because of economic consequences, but also because of significant health problems. For that reason, the determination of the authenticity of the food is a very important issue in quality control and food safety [1,2,3,4].

Drinks are products that can be very easily adulterated through practices that include mislabeling, supplementation with flavors or aromas, and the addition of unspecified additives to increase their volume, among other practices. Drinks with higher adulteration rates are fruit juices, coffee, tea, wine, and other alcoholic beverages [4].

Coffee is an infusion of ground roasted coffee beans from the coffee plant with a characteristic taste and aroma. Coffee is one of the most popular beverages in all the world, consumed by millions of people every day, occupying the second position of most commercialized basic products. Some 60 tropical and subtropical countries are coffee producers (with a world coffee production of 6.3 million tons per year), being for some of them, the main agricultural export product. The coffee plant belongs to *Coffea* from the *Rubiaceae* family, with more than 70 varieties, although the majority of the coffee consumed, so having major economic and commercial importance, is produced by the *Coffea Arabica* (Arabica) and *Coffea Canephora* (Robusta) varieties. The Arabica variety is the most appreciated by consumers and even considered better than the Robusta variety for its high sensorial properties. For this reason, Arabica coffee usually has a higher price in the international market [5,6]. Coffee is known as a stimulant, a property associated mainly to alkaloids such as caffeine to which the beneficial effects of coffee have been attributed for years. However, nowadays, it is known that this product also contains a high number of bioactive substances that contribute to the valuable properties of coffee, giving place to an important antioxidant activity. Coffee is rich in antioxidant substances like phenolic acids and polyphenols, being especially abundant ellagic, caffeic, and chlorogenic acids. Some studies relate its intake to the decrease in the development of some serious and prevalent diseases such as type II diabetes, liver cirrhosis, cancer, and some cardiovascular diseases [5,6]. The content of those substances, and consequently, the antioxidant characteristics of coffee can vary depending on the varieties, origin, roasting degree, and climatic conditions, among other factors [5,6,7].

Adulteration practices in roasted coffee are both frequent and diversified. They may be related not only to the quality of the coffee beans (their species, geographical origin, the use of defective coffee beans) [8,9,10,11,12], but also on the addition of other substances (coffee husks and stems, maize, barley, brown sugar, soybean, etc.) [13,14,15,16,17] to coffee blends to obtain an economical profit by making the final product less expensive. An interesting review addressing more than two decades of coffee adulteration was published by Toci et al. [18], revealing the need to control the authenticity and origin of coffee samples to prevent such bad practices. One of the most common coffee adulteration practices is that of Arabica with Robusta varieties blend [18,19,20,21]. A great variability of analytical techniques has also been reported during the last years to address the characterization and authentication of coffee, most of them in combination with chemometric procedures due to the huge amount of chemical data variables that can be obtained from the analyzed samples [18]. Both targeted and non-targeted approaches are proposed. For example, fingerprinting strategies using nuclear magnetic resonance (NMR) [14,22] or infrared (IR) [21,23] spectroscopy, as well as direct infusion-mass spectrometry [24], have been described to investigate adulterant contents in coffee samples. For the separation and quantification of bioactive substances in coffees to address adulteration practices, liquid chromatography (LC) with ultraviolet (UV) [13,25,26,27,28], fluorescence [29], and amperometric [17] detection, gas chromatography (GC) [11], and capillary electrophoresis [16,20,30] are widely used, in most cases in combination with mass spectrometry. For example, Daniel et al. [16] reported the monosaccharide profiling determination by capillary electrophoresis-mass spectrometry (CE-MS) to detect the adulteration of coffee samples with soybean and corn. 

This work will focus on the thematic of fraud detection in coffee. This manuscript offers an overall strategy to deal with the topic of food authentication, starting from simpler exploratory analyses to quantification of coffee adulterant percentages. In this work, a simple and efficient high-performance liquid chromatography with UV-detection (HPLC-UV) fingerprinting method was developed for the characterization, classification, and authentication of coffee samples according to the production regions, the coffee variety, and the roasting degree. Reversed-phase mode using a Kinetex^®^ C18 column (100 × 4.6 mm i.d., 2.6 µm particle size) under gradient elution conditions employing 0.1% formic acid aqueous solution and methanol as mobile phase components were proposed. Three hundred and six commercially available coffee samples divided into three sets of samples changing on the production country, coffee variety, and roasting degree were analyzed with the proposed methodology. The obtained HPLC-UV fingerprints were then employed as a source of chemical information to address the characterization and classification of the analyzed coffees by principal component analysis (PCA) and partial least squares regression-discriminant analysis (PLS-DA). Besides, HPLC-UV fingerprints were also evaluated as potential chemical descriptors to detect and quantify coffee sample adulterations by partial least squares regression (PLSR). 

Authentication of coffee origin (production country or region), as well as coffee varieties, is highly demanded by our society as an additional quality attribute, being willing to pay higher prices for coffee varieties produced in specific regions. In this context, simple and reliable methods to prevent fraudulent practices become necessary. For that purpose, the aim of this work focused on the evaluation of a non-targeted HPLC-UV fingerprinting method to address the classification and authentication of coffee samples according to three sets of coffee samples (described in Table 1). The first two sets explored variations regarding not only the coffee origin (country or region of production), but also the coffee variety (Arabica, Robusta) and its roasting degree. In contrast, the third set was designed to assess the applicability of the proposed method for the classification and authentication of coffee samples produced in nearby countries.

## 2. Materials and Methods 

### 2.1. Chemicals and Standard Solutions

For the mobile phase, methanol was obtained from PanReac AppliChem (HPLC grade, Barcelona, Spain) and formic acid (≥98%) from Sigma-Aldrich. An Elix 3 coupled to a Milli-Q system from Millipore Corporation (Burlington, MA, USA) was used to purify the water, filtering it through a 0.22 µm nylon membrane integrated into the Milli-Q system. Mineral water obtained from Eroski (Barcelona, Spain) was employed for brewing the coffees. 

### 2.2. Instrumentation

Chromatographic fingerprints were obtained on an HPLC instrument from Agilent HPLC 1100 Series (Waldbronn, Germany) equipped with a G1312A binary pump, a WPALS G1367A automatic sample injector, a G1315B diode-array detector, and a PC with the Agilent Chemstation software. A Kinetex^®^ C18 reversed-phase column (100 × 4.6 mm i.d., 2.6 µm particle size) provided by Phenomenex (Torrance, CA, USA) was used under gradient elution conditions employing 0.1% formic acid in water (*v*/*v*) (solvent A) and methanol (solvent B) as mobile phase components. The gradient started increasing the methanol component (solvent B) from 3% to 75% in 30 min. After that, there was an isocratic step of 2 min. Then, methanol increased from 75% to 95% in 2 min. Finally, the elution program came back to the initial composition in 0.2 min and an isocratic step of 5.8 min guaranteed column re-equilibration. The flow rate was 0.4 mL/min and the injection volume was 5 µL. UV acquisition was carried out at 280 nm.

### 2.3. Samples and Sample Treatment

A total of 306 commercially available coffee samples, grouped in three different sets, were analyzed. The first two sets, described in Table 1, comprised a total of 120 commercially available Nespresso^®^ coffee samples (each one) purchased from supermarkets in Barcelona (Spain). Coffee samples differed in the coffee variety (Arabica or Robusta), the region of origin, and the roasting degree (increasing from 1 to 5). The third set of samples included a total of 66 samples obtained from Vietnam and Cambodia supermarkets, and were classified into 5 groups depending on the coffee variety and the region of origin. No information regarding the roasting degree was available. 

Sample treatment consisted of coffee brewing with mineral water. In the case of the two first sets of samples, coffee was directly brewed by using an espresso machine (Nespresso), using in all cases the same brewing time to reach the same final volume. In the third set of samples, coffee beans were first grounded when necessary. Then, the coffees were brewed by employing an Italian coffee maker. For that purpose, ground coffee was introduced well compressed in the Italian coffee maker and 400 mL of mineral water was employed for the coffee brewing, with the help of a Bunsen burner to carry out the coffee lixiviation. All brewed coffees were then filtered with 0.45 µm nylon filters (Phenomenex) into 2 mL glass vials which were stored at −4 °C. Moreover, a quality control (QC) solution was prepared for every set of samples by mixing 50 µL of each sample extract. QCs were used to evaluate the repeatability of the method and the robustness of the chemometric results. 

**Figure 1:**
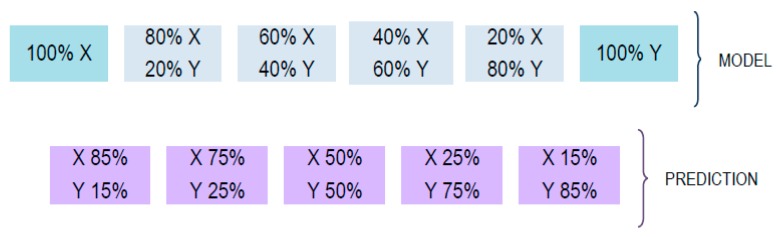
Coffee concentration levels employed in the adulteration studies (X original coffee sample, Y coffee sample used as adulterant).

Samples from the second and third sets were also employed for adulteration studies. For the second set, the adulteration cases proposed were Colombia vs. Ethiopia, Colombia vs. Nicaragua, and India vs. Indonesia. For the third set, the adulteration cases proposed were Vietnam-Arabica vs. Vietnam-Robusta, Vietnam-Arabica vs. Cambodia, and Vietnam-Robusta vs. Cambodia samples. For each adulteration case, two sets of samples were prepared for calibration (to build the PLSR model) and validation (for prediction purposes), with different amounts of adulterations as described in Figure 1.

The calibration set included the 20%, 40%, 60%, and 80% adulteration levels, as well as 100% pure coffees of each class, and the validation set included 15%, 25%, 50%, 75%, and 85% adulteration levels. Besides, an additional adulterated sample at a 50% level was employed as a QC solution. Each adulteration level (Figure 1) was prepared by quintuplicate obtaining a total of 55 sample extracts for each adulteration case. Similar calibration designs were used successfully elsewhere for predicting adulteration amounts [31,32]. Levels in this design were chosen to be realistic from the point of view of the authentication, since higher adulterant percentages could be of interest in fraudulent practices to obtain bigger illicit profits. 

### 2.4. Data Analysis

After sample treatment, all the samples were analyzed randomly with the proposed HPLC-UV method. The resulting fingerprints were used as sample chemical descriptors to build the different data matrices that were subjected to principal component analysis (PCA), partial least squares–discriminant analysis (PLS-DA), and partial least squares regression (PLSR) under SOLO 8.6 chemometric software from Eigenvector Research (Manson, WA, USA) [33]. Details of the theoretical background of these statistical methodologies are addressed elsewhere [34]. Indistinctly of the chemometric method to be used, X-data matrices consisted of the acquired HPLC-UV chromatographic fingerprints. Instead, Y-data matrices defined each sample class in PLS-DA, whereas it consisted of the adulterant percentage in PLSR. HPLC-UV fingerprints were autoscaled to provide the same weight to each variable by suppressing differences in their magnitude and amplitude scales. The most appropriate number of latent variables (LVs), in PLS-DA and PLSR, was established by considering the first significant minimum point of the cross-validation (CV) error from a Venetian blind approach. The validation of the PLS-DA models was carried out by using 70% of samples as the calibration set, while the remaining 30% constituted an independent validation set. In the case of PLSR, models were validated on the prediction sets as defined in Figure 1.

## 3. Results and Discussion

### 3.1. HPLC-UV Method

The main objective of this section was to achieve a simple non-targeted HPLC-UV fingerprinting method for the classification and authentication of coffee samples. In this regard, an adequate chromatographic elution program to obtain good HPLC-UV fingerprints was optimized, taking into consideration that polyphenols and related compounds are quite abundant in coffee and they could serve as the basis of sample discriminations (see Table S1, Supplementary Material). Their chromatographic separation was evaluated by reversed-phase chromatography using a porous-shell Kinetex C18 column, and using acidified aqueous solutions and methanol as the mobile phase components, as typically described in the literature [35]. Therefore, as a first approach, the separation of 15 polyphenol and phenolic acid compounds was attempted using a universal elution gradient profile (from 5% to 95% methanol in 30 min), and then back to initial conditions for column re-equilibration. Under these circumstances, all studied compounds were retained in the C18 column and detected, although full baseline separation for all of them was not achieved, and numerous partial co-elutions were observed mainly at the beginning of the chromatogram. Hence, the chromatographic separation was optimized by applying different elution profiles combining both linear gradient and isocratic steps. Optimal chromatographic elution program (see Instrumentation section) was selected as a compromise between the separation of the 15 studied compounds and total analysis time and taking into consideration that the focus was not on targeted analysis but on non-targeted HPLC-UV fingerprinting. Figure S1 shows the HPLC-UV chromatogram obtained at 280 nm when a standard solution of the 15 polyphenol and phenolic acid compounds (each at 20 mg/L) was analyzed under the optimized gradient program. Despite several coelutions (homovanillic and chlorogenic acids, peaks 3−4; syringic acid and vanillin, peaks 6−7; and ferulic and veratric acids, peaks 12−13), it was acceptable for the acquisition of HPLC-UV fingerprints of coffee. Therefore, these chromatographic separation conditions were selected for the non-targeted analysis of the studied coffee samples.

### 3.2. Non-Targeted HPLC-UV Fingerprints of Coffees

**Figure 2:**
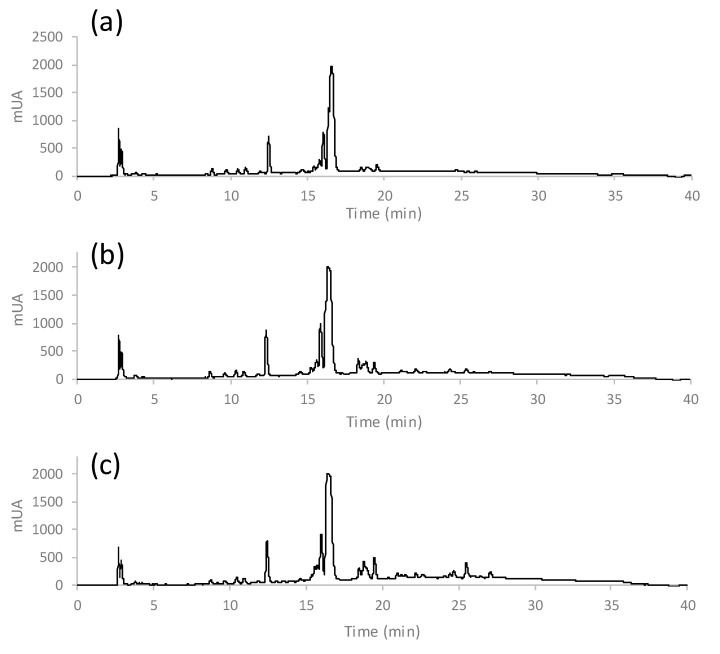
Non-targeted high-performance liquid chromatography with ultraviolet detection (HPLC-UV) fingerprints at 280 nm of three coffees of the first set of samples. (**a**) Arabica coffee from Ethiopia, (**b**) Arabica-Robusta mixture coffee from India, (**c**) Robusta coffee from Uganda.

A total of 306 commercially available coffee samples (grouped in three sets as previously described, Table 1) were analyzed with the proposed method and the corresponding HPLC-UV fingerprints of the brewed coffee samples were registered at 280 nm. As an example, Figure 2 shows the chromatograms of three coffees belonging to the first set of samples, an Arabica coffee from Ethiopia (**a**), an Arabica-Robusta mixture coffee from India (**b**), and a Robusta coffee from Uganda. 

Additional examples of fingerprints for coffees belonging to the other two sets of samples are included in Figures S2 and S3 (Supplementary Material). As can be seen in the figures, noteworthy differences in terms of number and abundance of the detected compounds (considering the retention time), as well as the peak intensities, were obtained. Besides, since fingerprints were reproducible among samples belonging to the same coffee class, they were proposed and evaluated as chemical descriptors to address sample classification by multivariate chemometric methods. 

### 3.3. Sample Exploration by PCA 

**Figure 3:**
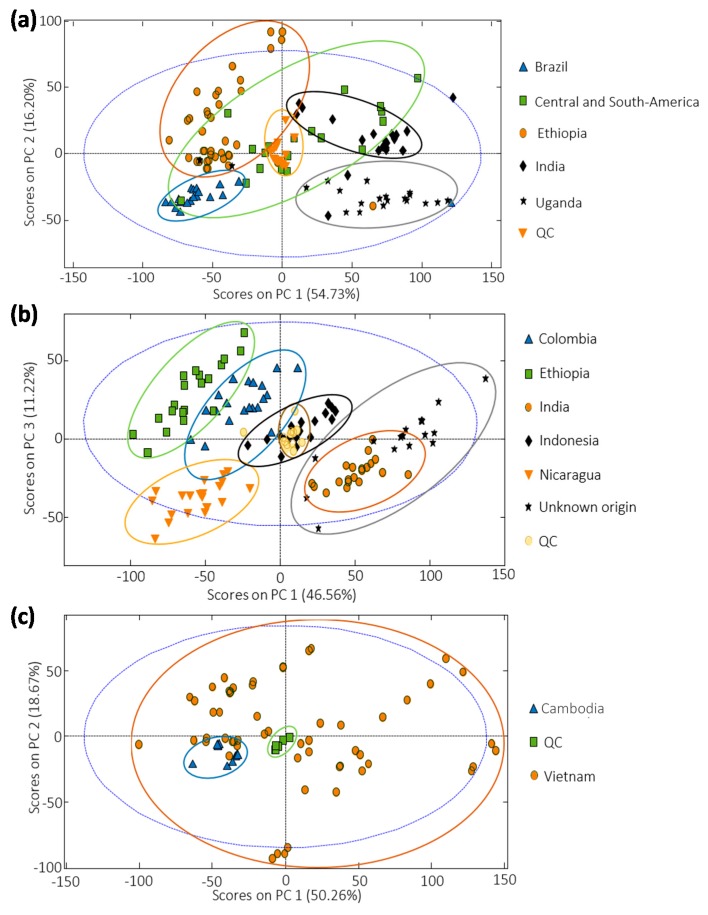
Scatter plot of scores for (**a**) Sample set 1 (PC1 vs. PC2), (**b**) Sample set 2 (PC1 vs. PC3), and (**c**) Sample set 3 (PC1 vs. PC2).

The capability of HPLC-UV fingerprints to be used as discriminant chemical descriptors for coffee sample classification according to the different regions of origin was first evaluated by PCA. This study was also aimed to assess the QC samples’ behavior. For that purpose, X-data matrices for each set of samples (including the corresponding QCs) consisting of the absorbance signals recorded as a function of retention time were built, with dimensions of 133 × 6001, 133 × 6001, and 72 × 6000 for sample sets 1, 2, and 3, respectively. Besides, autoscaling pretreatment was chosen to provide similar weight to all the variables. Figure 3 shows the best 2D PCA score plots obtained for each set of samples. As can be seen, QCs appeared well grouped in the center of each plot showing the good performance and reproducibility of the proposed method and evidencing the validity of the chemometric results. 

Regarding coffee distribution, samples tend to be grouped according to their region of origin, although overlapping of several groups of samples can be observed. The best discrimination was achieved with the coffee set of samples 2 (Figure 3b), where acceptable discrimination for almost all the sample groups was achieved except for coffees from India and those of unknown origin. In the case of coffee samples produced in nearby countries (Figure 3c for Vietnamese and Cambodian coffees), no group discrimination was observed by PCA, although a trend can be observed, being Cambodian samples quite grouped at the bottom-left area of the plot. 

### 3.4. Sample Classification by PLS-DA

**Figure 4:**
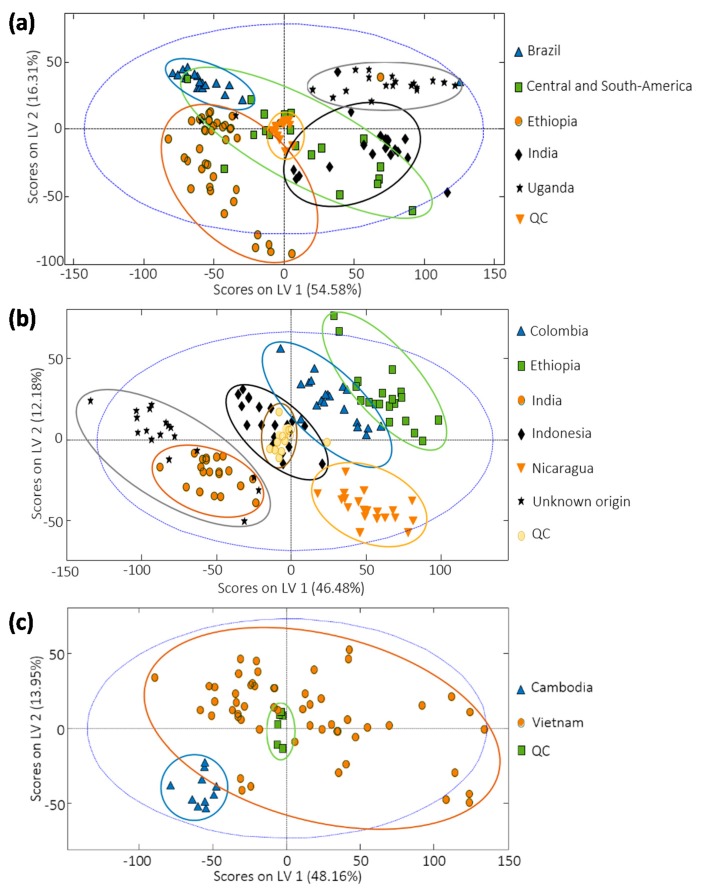
Partial least square regression-discriminant analysis (PLS-DA) score plots of LV1 vs. LV2 for (**a**) coffee set of samples 1, (**b**) coffee set of samples 2, and (**c**) coffee set of samples 3, when using chromatographic fingerprints to classify coffee samples according to their region of origin.

HPLC-UV chromatographic fingerprints were also used to address coffee classification by using PLS-DA. In this case, the classification of coffee samples was addressed as a function of three factors: the region of origin, the coffee variety (Arabica vs. Robusta), and the roasting degree. Figure 4 depicts the best PLS-DA score plots obtained for the three coffee sets of samples under study for the classification according to their region of origin. Best models were established with 4 LVs as deduced by CV based on the Venetian blind approach. 

Regarding the sample distribution as a function of the coffee region of origin, the classification method generally improves in comparison to the previous PCA results, as expected, although again overlapping between some groups is observed. Considering the coffee set of samples 1 (Figure 4a), the overlapping of the coffee samples labeled as Central and South America with other groups remains. This could be attributed to the possible composition of these coffees (a mixture of coffees from different countries from Central and North America region), giving place to confusion with other groups of samples. Considering the other classes, full discrimination among them was accomplished. In the case of the coffee set of samples 2, classification and sample discrimination also improved with PLS-DA (Figure 4b), especially between samples from Ethiopia and Colombia, and between Indonesia and both India and Unknown origin, in comparison to PCA (Figure 3b). Again, samples of unknown origin are grouped with the coffees cultivated in India, as was also observed by PCA. Finally, results improved considerably when addressing coffee samples produced in nearby countries, such as the case of coffees from Vietnam and Cambodia (Figure 4c). By employing PLS-DA, Cambodian samples were completely separated from Vietnamese samples. 

In a similar way, Figures S4 and S5 (Supplementary Material) show the corresponding PLS-DA score plots obtained for the analyzed coffee samples as a function of the coffee variety (Arabica, Robusta, and mixtures) and the coffee roasting degree, respectively. As can be seen in Figure S4, HPLC-UV fingerprints seem to be also good descriptors to address sample classification according to the coffee variety. LV1 and LV2 are mainly responsible for this discrimination. In the case of the coffee set of samples 1, 100% Robusta samples are perfectly grouped and separated from the other samples at the top-right area of the score plot. Then, 100% Arabica samples are distributed on the bottom-left area while Arabica-Robusta mixture samples located in between. In the case of the coffee set of samples 2, the behavior was similar, with two groups perfectly discriminated, corresponding to 100% Arabica at the right and Arabica-Robusta mixture samples at the left. A certain trend is also observed when focusing on the coffee set of samples 3, with Robusta samples distributed at the top-right area of the plot, and the Arabica samples at the bottom-left area, although overlapping is observed. However, it should be noted that in this case, only Vietnamese samples were considered in the PLS-DA model (Figure S4c) because no information regarding Cambodian coffee varieties was available. 

Regarding coffee sample classification according to the roasting degree (Figure S5), only PLS-DA models for the coffee set of samples 1 and 2 were built, because no information on this parameter was available for Vietnamese and Cambodian coffee samples. From both PLS-DA score plots, it was found that samples were distributed according to the coffee roasting degree as a function of both LV1 and LV2. In the case of the coffee set of samples 1 (Figure S5a), the less roasted coffees (1/5 roasting degree) were located at the bottom-left area of the plot, exhibiting low score values on both LV1 and LV2, while those exhibiting higher roasting degrees occupied the top-right area, depicting high LV1 and LV2 score values. As regards to the coffee set of samples 2 (Figure S5b), locations of the samples depending on the roasting factor followed a similar pattern, with the less roasted samples clustered at the left part of the plot (low LV score values), and with the most roasted coffee samples (5/5 roasting degree) at the top-right area (high LV score values).

These results demonstrate that chromatographic fingerprints can be proposed as good descriptors to address coffee samples characterization and classification according to the coffee region of origin (production country), the coffee variety (Arabica vs. Robusta), and the coffee roasting degree. 

### 3.5. Supervised PLS-DA Method Validation

**Figure 5:**
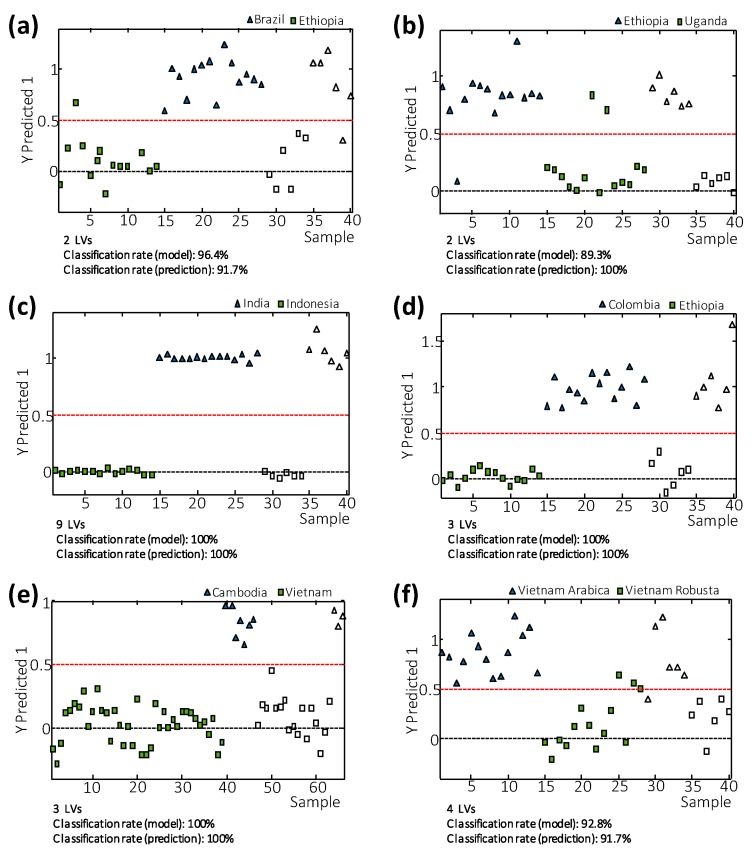
Sample vs. Y predicted 1 values plot for (**a**) Brazilian vs. Ethiopian coffees, (**b**) Ethiopian vs. Uganda coffees, (**c**) Indonesian vs. Indian coffees, (**d**) Colombian vs. Ethiopian coffees, (**e**) Vietnamese vs. Cambodian coffees, and (**f**) Vietnamese Arabica vs. Vietnamese Robusta coffees. Filled and empty symbols correspond to calibration and validation sets, respectively. Dashed red lines represented the threshold separating one class from the other. The number of latent variables (LVs) employed to generate each classificatory model and sample classification rate are also indicated.

To demonstrate the applicability of the proposed methodology based on HPLC-UV fingerprinting, the classification rate was studied for some paired PLS-DA models: (i) Brazilian vs. Ethiopian coffees, (ii) Ethiopian vs. Uganda coffees, (iii) Indonesian vs. Indian coffees, (iv) Colombian vs. Ethiopian coffees, (v) Vietnamese vs. Cambodian coffees, and (vi) Vietnamese Arabica vs. Vietnamese Robusta coffees. For that purpose, each paired PLS-DA chemometric model studied was established using 70% of each sample group (selected randomly) as the calibration set, while the remaining 30% of the samples were employed as an “unknown” set of samples for validation purposes. This means that except for Vietnamese vs. Cambodian coffees (case v), the rest of the models were created with 24 calibration samples (14 of each class) while models were applied to assign 12 unknown samples (6 of each type). In the case v, Vietnamese and Cambodian standards were 39 and 17, respectively, and the prediction set was composed of 7 Vietnamese and 3 Cambodian coffees. As examples, Figure 5 shows the classification plots for the six paired PLS-DA models studied. The number of LVs to generate each classificatory model is also indicated in the figure. Dashed red lines represented the threshold, separating one class from the other, thus samples were at the top or bottom of these lines depending on their class memberships.

As can be seen, in most cases, 100% sample classification rates for both calibrations and predictions are obtained. In the case of Brazilian vs. Ethiopian coffees (Figure 5a) and Ethiopian vs. Ugandan coffees (Figure 5b), the sample classification rates for calibration were 96.4% and 89.3%, respectively, and only one sample used as unknowns was not correctly classified in the case of Brazilian vs. Ethiopian coffees. When comparing Vietnamese coffee varieties (Arabica vs. Robusta) (Figure 5f), sample classification rate for prediction was 91.7% (only one sample used as an unknown was not correctly assigned). Therefore, it seems that the HPLC-UV fingerprints may be used for authentication purposes in the prevention of coffee fraud.

### 3.6. Quantitation of Coffee Adulterations by PLSR

**Figure 6:**
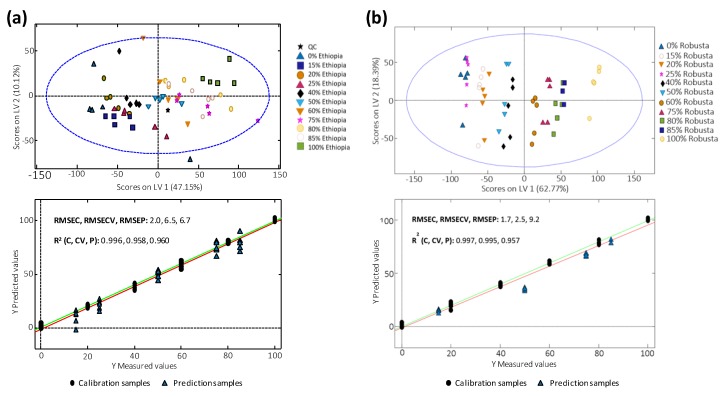
LV1 vs. LV2 score plot of both calibration and validation sets by PLSR for (**a**) Colombian coffee adulterated with Ethiopian coffee and (**b**) Vietnamese Arabica coffee adulterated with Vietnamese Robusta coffee. Models (**a**,**b**) were created with 6 and 4 LVs, respectively.

The capability of fingerprints to quantify the extension of coffee adulterations was evaluated by PLSR. For that purpose, several adulteration cases were studied: (i) Colombian coffee adulterated with Ethiopian coffee, (ii) Colombian coffee adulterated with Nicaraguan coffee, (iii) Indian coffee adulterated with Indonesian coffee, (iv) Vietnamese Arabica coffee adulterated with Vietnamese Robusta coffee, (v) Vietnamese Arabica coffee adulterated with Cambodian coffee, and (vi) Vietnamese Robusta coffee adulterated with Cambodian coffee. For each adulteration case, two independent sets of samples were available for calibration and validation, as described in Figure 1. As examples, Figure 6 shows the distribution of all the adulteration levels in both calibration and validation sets in the space of LV1 vs. LV2 as well as the PLSR multivariate calibration model obtained for (**a**) Colombian coffee adulterated with Ethiopian coffee and (**b**) Vietnamese Arabica coffee adulterated with Vietnamese Robusta coffee. The distribution of these samples on LV1 and LV2 depended on both chromatographic features and adulterant concentrations which increased, in general, from the bottom-left to the top-right part. Results related to all the other adulteration cases studied are depicted in Figure S6. Root mean square errors of calibration (RMSEC), cross-validation (RMSECV), and prediction (RMSEP), as well as the corresponding determination coefficients (R^2^), are also indicated in the figures.

As can be seen in the PLSR models built according to the contents of the coffee acting as an adulterant, 100% pure coffee samples tend to be located in the opposite areas of the score plots, mainly showing either a positive or a negative correlation with respect LV1. The optimal numbers of LVs deduced from cross-validation were 6 and 4 for Colombian coffee adulterated with Ethiopian coffee (Figure 6a) and Vietnamese Arabica coffee adulterated with Vietnamese Robusta coffee (Figure 6b), respectively. Taking into account the complexity of the data regarding compositional and chromatographic issues, we guessed that these numbers or LVs were reasonably low to avoid overfitting problems. Then, the adulterated coffee samples are distributed in between the 100% pure coffee samples according to the coffee adulterant level. For example, 100% pure Vietnamese Arabica coffee is located in the left part of the score plot (Figure 6b), while 100% pure Vietnamese Robusta coffee is in the right part. Then, the adulterated samples are distributed from left to right according to the increase in the Robusta coffee adulteration level. It should be noted that small variations in the general clustering distribution trend are observed when close adulterant levels are employed, such as in the case of 40−50% or 15−20−25% Robusta adulterant contents, probably because in those cases, LV2 is also influencing the sample group classification and distribution. This behavior is generally also observed in all the other cases studied (Figure 6a and Figure S6).

In general, good results were obtained when PLSR multivariate calibration was employed for the quantitation of adulterant levels in coffee, obtaining R^2^ coefficients in calibration, cross-validation, and prediction models higher than 0.957, showing the good performance of the proposed methodology. There is only one exception, which is the case of the Colombian coffee adulterated with a Nicaraguan coffee (Figure S6a), where although a very good correlation coefficient was obtained for calibration, values for cross-validation and prediction worsened considerably with R^2^ of 0.391 and 0.661, respectively. RMSEC, RMSECV, and RMSEP values were also, in general, acceptable in most of the evaluated cases, with values below 2.9%, 6.5%, and 8.9%, respectively. Again, except for the Colombian coffee adulterated with Nicaraguan coffee that showed RMSECV and RMSEP values of 27.9% and 18.3%, respectively. It should be also noted that the prediction errors obtained for the Vietnamese Arabica and the Vietnamese Robusta coffees, both of them adulterated with a Cambodian coffee, are remarkably low (RMSEP values of 4.5%), taking into consideration that these coffees are produced in very nearby countries, with very similar weather conditions. Obviously, other parameters such as the coffee variety are also contributing to this good sample prediction.

## 4. Conclusions

In the present work, non-targeted HPLC-UV fingerprints acquired at 280 nm have proved to be acceptable sample chemical descriptors for the characterization, classification, and authentication of coffee samples according to their country of production, variety, and roasting degree, even in the cases of coffees produced in nearby countries such as Vietnam and Cambodia. HPLC-UV fingerprinting descriptors were easily obtained by reversed-phase chromatography using a conventional C18 column directly after brewing the coffee without any sample treatment other than filtration, thus reducing considerably sample manipulation. 

Exploratory analysis by PCA and PLS-DA using the obtained coffee HPLC-UV fingerprints showed, in general, good discrimination capabilities among the different coffee production regions, coffee varieties (Arabica vs. Robusta), and coffee roasting degrees. PLS-DA provided good classification rates for most of the studied examples, always higher than 89.3% and 91.7% for PLS-DA calibration and prediction, respectively. 

The capability of the proposed methodology to detect and quantify coffee frauds (down to 15% adulterant level) using PLSR multivariate calibration was evaluated by studying several adulteration cases involving coffees adulterated either with those produced in a different country or of a different coffee variety. Very acceptable calibration, cross-validation, and prediction errors, with values lower than 2.9%, 6.5%, and 8.9%, respectively, were obtained for most of the evaluated cases.

Therefore, the proposed non-targeted HPLC-UV fingerprinting methodology resulted to be a feasible, simple, and cheap methodology to address coffee authentication, especially for developing coffee production countries.

## Figures and Tables

**Table 1:** Description of the analyzed coffee samples.

Commercial Name	Number of Samples	Coffee Variety	Origin Region	Roasting Degree
-	-	**Set of sample 1**	-	-
Arabica Ethiopia Harrar	20	Arabica	Ethiopia	1/5
Bukeela	20	Arabica-Arabica Mixture	Ethiopia	1/5
Dulsao	20	Arabica	Brazil	2/5
Arpeggio	20	Arabica	Central and South-America	4/5
Indriya	20	Arabica-Robusta Mixture	India	4/5
Robusta Uganda	20	Robusta	Uganda	4/5
-	-	**Set of sample 2**	-	-
Master Origin Colombia	20	Arabica	Colombia	3/5
Master Origin Ethiopia	20	Arabica	Ethiopia	2/5
Master Origin India	20	Arabica-Robusta Mixture	India	5/5
Master Origin Nicaragua	20	Arabica	Nicaragua	2/5
Master Origin Indonesia	20	Arabica	Indonesia	4/5
Paris Black	20	Arabica-Robusta Mixture	Unknown origin	4/5
-	**-**	**Set of sample 3**	-	-
-	20	Arabica	Vietnam	Unknown
-	20	Robusta	Vietnam	Unknown
-	10	Mixture	Vietnam	Unknown
-	6	Unknown	Vietnam	Unknown
-	10	Unknown	Cambodia	Unknown

## Supplementary Materials

The following are available online at https://www.mdpi.com/2304-8158/9/3/378/s1, Table S1: Polyphenol and phenolic acid compounds used for optimizing the HPLC-UV separation, Figure S1: HPLC-UV chromatogram (at 280 nm) obtained with a standard solution of 15 polyphenol and phenolic acid compounds (each at 20 mg/L). Peak identification as in Table S1. Figure S2: HPLC-UV Fingerprints (at 280 nm) of three coffees of the second set of samples. (**a**) Arabica coffee from Colombia, (**b**) Arabica coffee from Nicaragua, and (**c**) Arabica-Robusta mixture coffee from an unknown origin. Figure S3: HPLC-UV Fingerprints (at 280 nm) of three coffees of the third set of samples. (**a**) Arabica coffee from Vietnam, (**b**) Robusta coffee from Vietnam, and (**c**) coffee from Cambodia. Figure S4: PLS-DA score plots of LV1 vs. LV2 for (**a**) coffee set of samples 1, (**b**) coffee set of samples 2, and (**c**) coffee set of samples 3, when using chromatographic fingerprints as chemical descriptors of coffee variety (Arabica, Robusta, or mixtures). A total of 4, 2, and 2 LVs for sets 1, 2, and 3, respectively, were established. Figure S5: PLS-DA score plots of LV1 vs. LV2 for (**a**) coffee set of samples 2, and (**b**) coffee set of samples 3, when using chromatographic fingerprints as chemical descriptors of the coffee samples according to their roasting degree (1/5 lowest to 5/5 highest roasting degree). A total of 2 LVs for all the sample sets were established. Figure S6: LV1 vs. LV2 score plot of the adulteration levels employed in both calibration and validation sets, and PLSR predictions for (**a**) Colombian coffee adulterated with Nicaraguan coffee (model with 3 LVs), (**b**) Indian coffee adulterated with Indonesian coffee (model with 4 LVs), (**c**) Vietnamese Arabica coffee adulterated with Cambodian coffee (model with 6 LVs), and (**d**) Vietnamese Robusta coffee adulterated with Cambodian coffee (model with 4 LVs).
