# Supplementary material for: Authentication of the Origin, Variety and Roasting Degree of Coffee Samples by Non-Targeted HPLC-UV Fingerprinting and Chemometrics. Application to the Detection and Quantitation of Adulterated Coffee Samples
Source: Foods. 2020 Mar 24;9(3):378. doi: 10.3390/foods9030378 (PMC7142590; doi:10.3390/foods9030378)
Supplement: Supplementary file 1 [file foods-09-00378-s001.pdf]

Supplementary Material

Authentication of the Origin, Variety and Roasting Deagree of Coffee Samples by Non-Targeted HPLC-UV Fingerprinting and Chemometrics. Application to the Detection and Quantitation of Adulterated Coffee Samples.

Nerea Núñez [1,*], Xavi Collado [1], Clara Martínez [1], Javier Saurina [1,2] and Oscar Núñez [1,2,3,*]

[1] Department of Chemical Engineering and Analytical Chemistry, University of Barcelona, Martí i Franquès 1-11, E08028 Barcelona, Spain; nereant7@gmail.com (N.N.); xavi.collado08@gmail.com (X.C.); clmartig28@alumnes.ub.edu (C.M.); xavi.saurina@ub.edu (J.S.); oscar.nunez@ub.edu (O.N.)

[2] Research Institute in Food Nutrition and Food Safety, University of Barcelona, Recinte Torribera, Av. Prat de la Riba 171, Edifici de Recerca (Gaudí), Santa Coloma de Gramenet, E08921 Barcelona, Spain

[3] Serra Húnter Fellow, Generalitat de Catalunya, Rambla de Catalunya 19-21, E08007 Barcelona, Spain

* Correspondence: nereant7@gmail.com (N.N.); oscar.nunez@ub.edu (O.N.); Tel.: +34-93-403-3706

Table S1. Polyphenol and phenolic acid compounds used for optimizing the HPLC-UV separation.

Peak	Compound	Family	CAS Number	Structure
1	**Homogentisic acid**	Phenolic acid	451-13-8	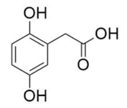
2	**(+)-Catechin hydrate**	Flavanol	225937-10-0	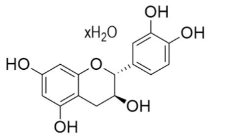
3	**Homovanillic acid**	Phenolic acid	306-08-1	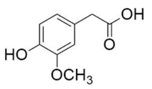
4	**Chlorogenic acid**	Phenolic acid	327-97-9	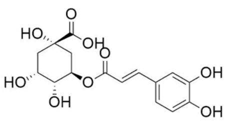
5	**Caffeic acid**	Phenolic acid	331-39-5	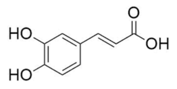

**6**	**Syringic acid**	Phenolic acid	530-57-4	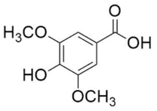
**7**	**Vanillin**	Phenolic aldehyde	121-33-5	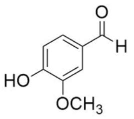
**8**	**Syringaldehyde**	Phenolic aldehyde	134-96-3	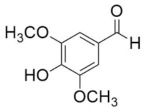
**9**	**(-)- Epicatechin**	Flavanol	490-46-0	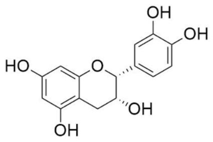
**10**	***p*-Coumaric acid**	Phenolic acid	501-98-4	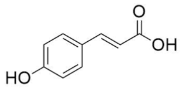
**11**	**Polydatin**	Stillbene	27208-80-6	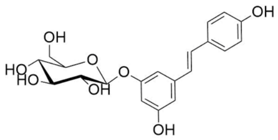
**12**	**Ferulic acid**	Phenolic acid	537-98-4	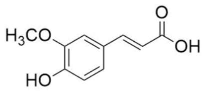
**13**	**Veratric acid**	Phenolic acid	93-07-2	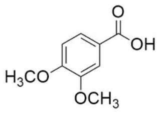
**14**	**Quinic acid**	-	77-95-2	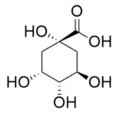
**15**	**Fisetin**	Flavanol	528-48-3	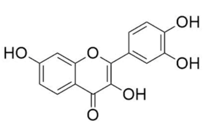

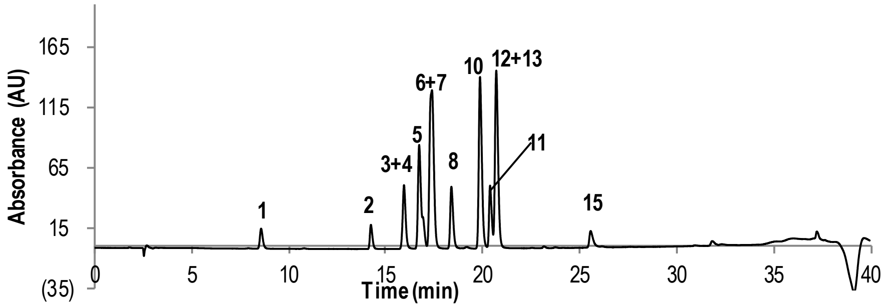

Figure S1. HPLC-UV chromatogram (at 280 nm) obtained with a standard solution of 15 polyphenol and phenolic acid compounds (each at 20 mg/L). Peak identification as in Table S1.

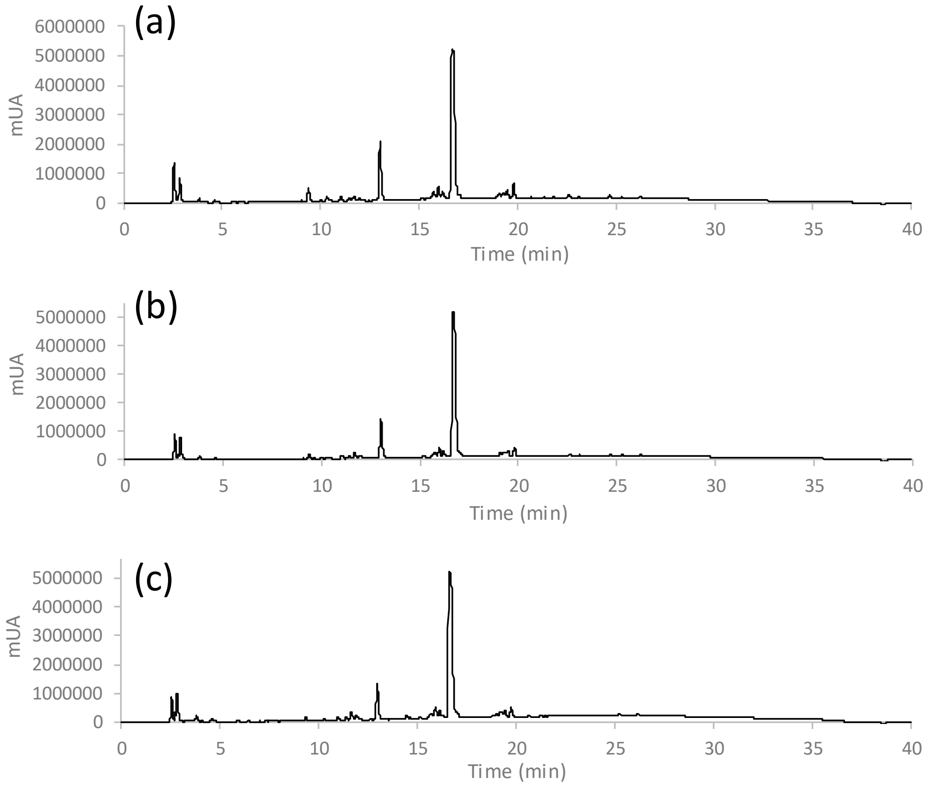

Figure S2. HPLC-UV Fingerprints (at 280 nm) of three coffees of the second set of samples. (a) an Arabica coffee from Colombia, (b) an Arabica coffee from Nicaragua, and (c) an Arabica-Robusta mixture coffee from an Unknown origin.

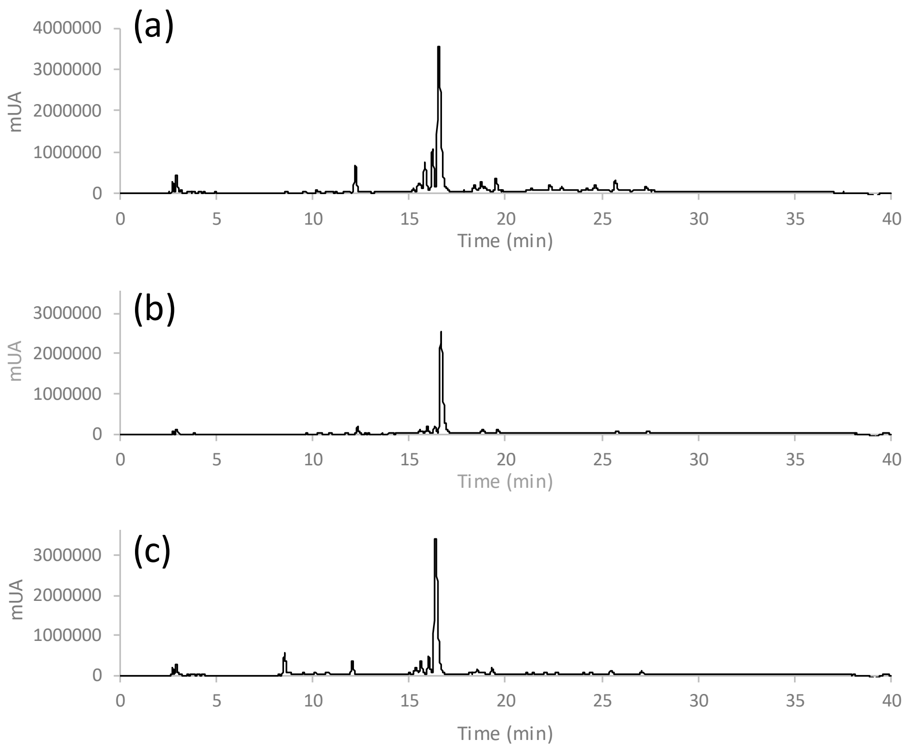

Figure S3. HPLC-UV Fingerprints (at 280 nm) of three coffees of the third set of samples. (a) an Arabica coffee from Vietnam, (b) a Robusta coffee from Vietnam, and (c) a coffee from Cambodia.

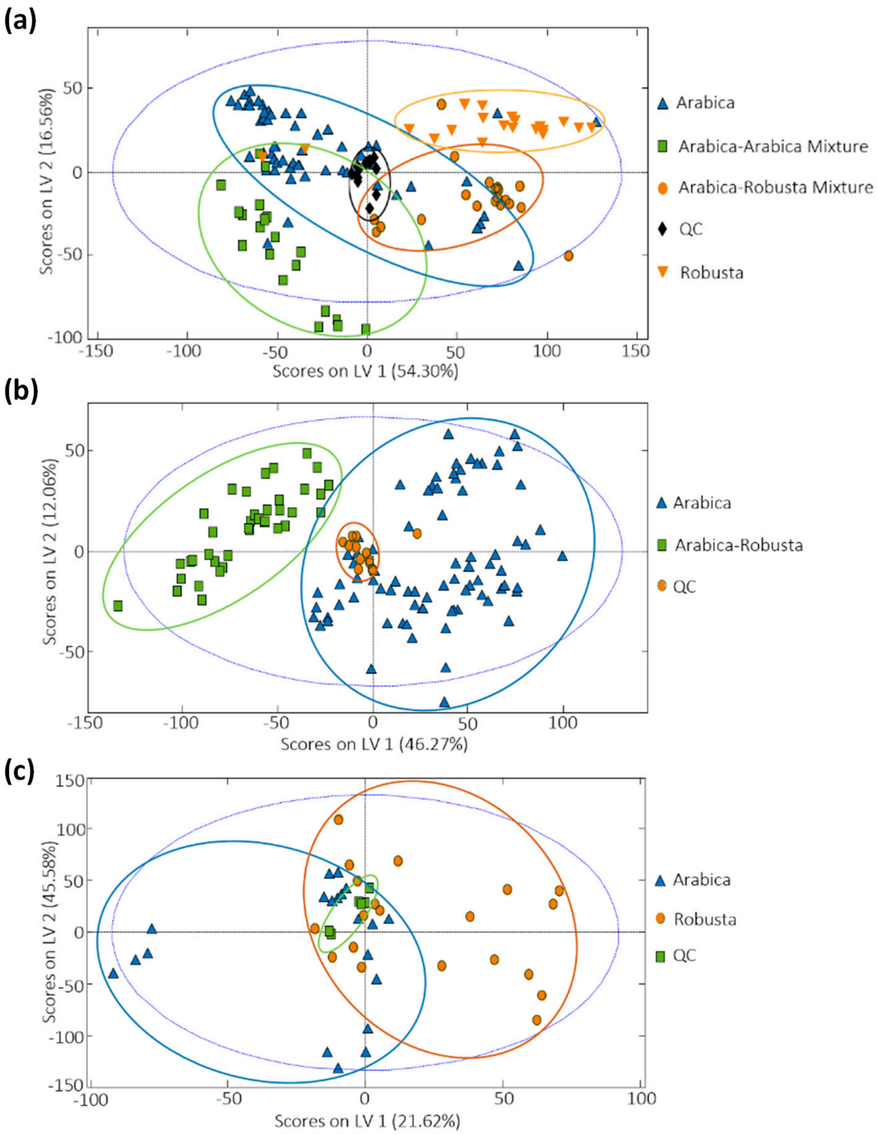

Figure S4. PLS-DA score plots of LV1 vs. LV2 for (a) coffee set of samples 1, (b) coffee set of samples 2, and (c) coffee set of samples 3, when using chromatographic fingerprints as chemical descriptors of coffee variety (Arabica, Robusta or mixtures). A total of 4, 2, and 2 LVs for sets 1, 2 and 3, respectively, were established.

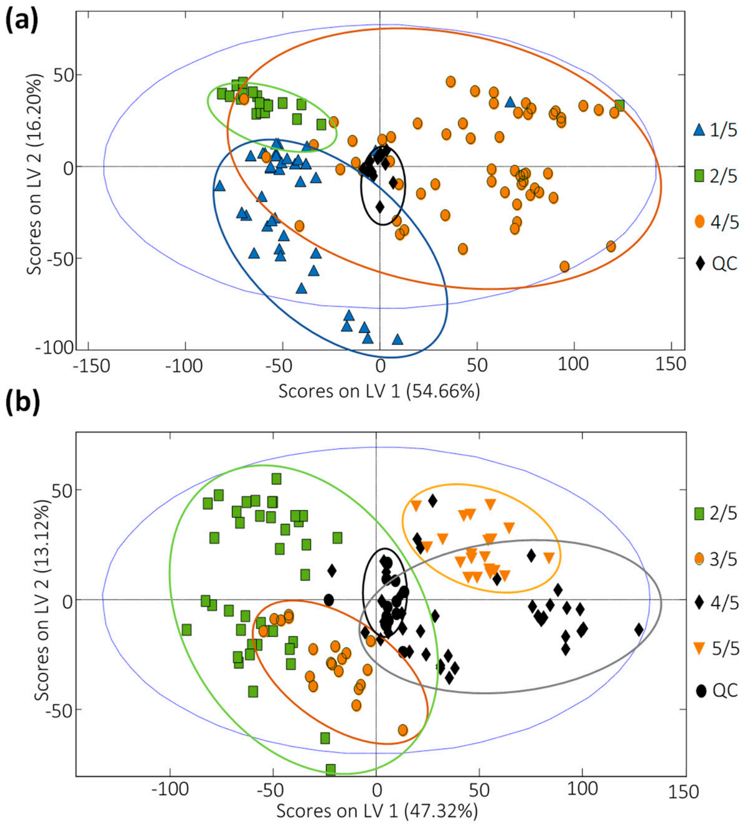

Figure S5. PLS-DA score plots of LV1 vs. LV2 for (a) coffee set of samples 2, and (b) coffee set of samples 3, when using chromatographic fingerprints as chemical descriptors of the coffee samples according to their roasting degree (1/5 lowest to 5/5 highest roasting degree). A total of 2 LVs for all the sample sets were established.

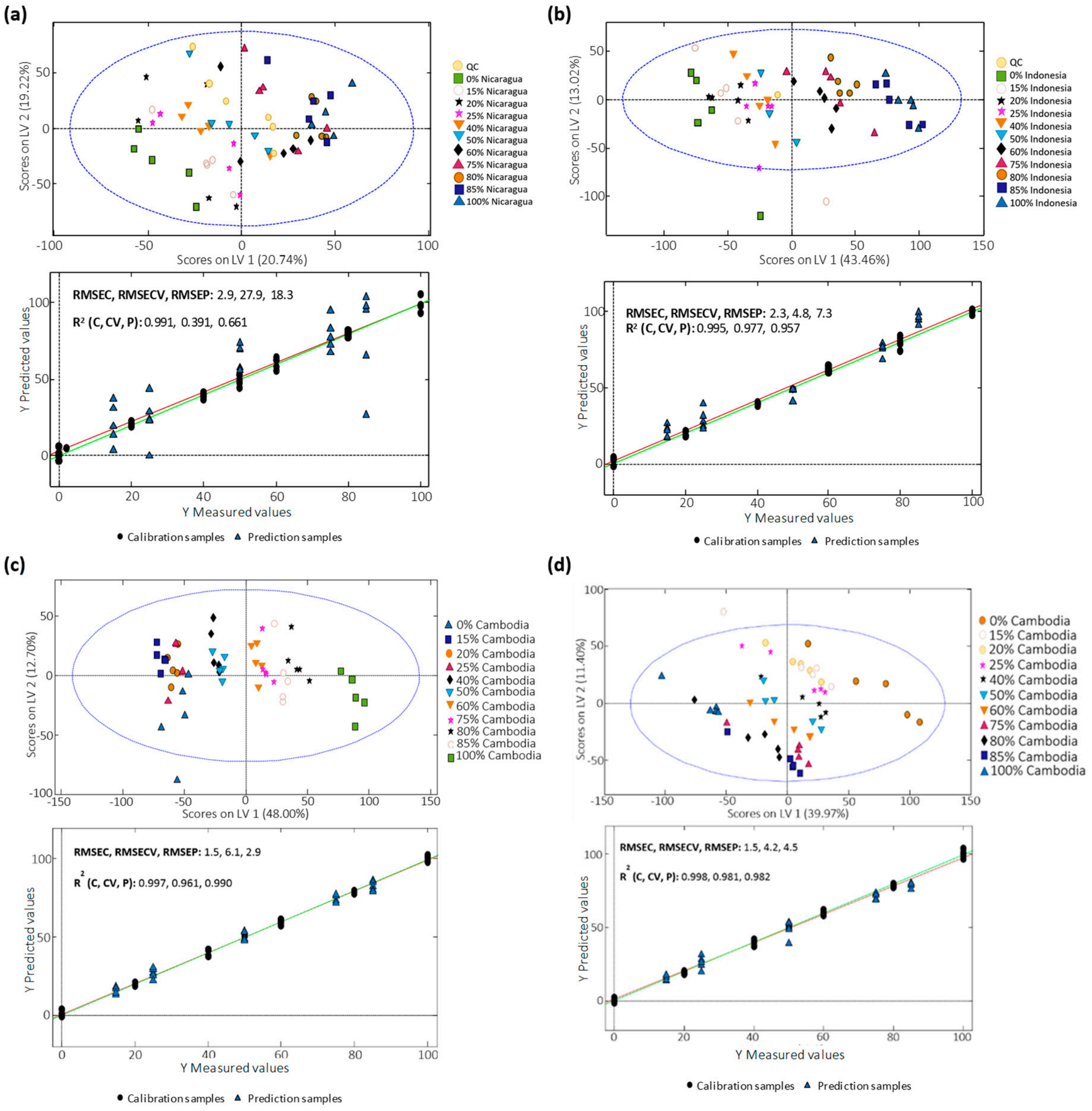

Figure S6. LV1 vs. LV2 score plot of the adulteration levels employed in both calibration and validation sets, and PLSR predictions for (a) Colombian coffee adulterated with Nicaraguan coffee (model with 3 LVs), (b) Indian coffee adulterated with Indonesian coffee (model with 4 LVs), (c) Vietnamese Arabica coffee adulterated with Cambodian coffee (model with 6 LVs), and (d) Vietnamese Robusta coffee adulterated with Cambodian coffee (model with 4 LVs).
